# An equity analysis of remote patient monitoring programs unveils assumptions on digital health equity

**DOI:** 10.1038/s41746-025-01731-x

**Published:** 2025-05-29

**Authors:** Ibukun-Oluwa Omolade Abejirinde, Vanessa Kishimoto, Kaylen J. Pfisterer, Nishath Uddin, Katherine McGuire, Janette Brual, Kaitlyn Merriman, Payal Agarwal, Jeanette Smith, Myrtede Alfred, Michele Strom, Quynh Pham

**Affiliations:** 1 Women’s College Hospital, Institute for Health System Solutions and Virtual Care, Toronto, ON Canada; 2 Dalla Lana School of Public Health, University of Toronto, Toronto, ON Canada; 3 Institute for Better Health, Trillium Health Partners, Mississauga, ON Canada; 4 Centre for Digital Therapeutics, University Health Network, Toronto, ON Canada; 5 Systems Design Engineering, University of Waterloo, Waterloo, ON Canada; 6 Department of Psychology, Toronto Metropolitan University, Toronto, ON Canada; 7 Gerstein Science Information Centre, University of Toronto, Toronto, ON Canada; 8 Department of Family and Community Medicine, University of Toronto, Toronto, ON Canada; 9 Patient Partner Evaluator, Chatham, ON Canada; 10 Department of Mechanical and Industrial Engineering, University of Toronto, Toronto, ON Canada; 11 Institute of Health Policy, Management and Evaluation, University of Toronto, Toronto, ON Canada; 12 Telfer School of Management, University of Ottawa, Ottawa, ON Canada; 13 School of Public Health Sciences, University of Waterloo, Waterloo, ON Canada

**Keywords:** Health care, Social sciences

## Abstract

To assess if remote patient monitoring (RPM) programs for chronic conditions are positioned to advance equity through inclusive practices, 119 papers were evaluated against 11 multi-level equity parameters. Equity parameters were inconsistent and incompletely reported suggesting assumptions are being made about the inherent equitability of RPM solutions. Reporting guidelines should include how RPM programs address inclusive strategies. A validated monitoring tool is needed to quantitatively assess equity progress and gaps.

## Introduction

In the last two decades, digital technologies have been leveraged to support remote patient monitoring (RPM). RPM is the use of digitally-mediated services for continuous or regular monitoring of a patients physical and physiological state to facilitate timely detection and action at the onset or early deterioration of illness^1–3^. At a systems level, RPM is often positioned to potentially free up limited acute care resources and improve the quality of life for sub-acute patients (i.e., patients with conditions that are long-lasting, but not chronic) who are clinically eligible for alternate service delivery models. Indeed, RPM applications serve various clinical programs including chronic care (e.g., heart failure, diabetes, hypertension etc.), and post-surgical management. Particularly during the COVID-19 pandemic, RPM gained prominence when public health measures and strained demands on the health care system mandated rapid uptake of digital solutions to support continuity of care from a distance^4,5^.

With projected increases in health care demand and utilization globally, it is anticipated that like other digital health technologies, RPM will play a role in supporting self-management and continuity of care for several disease domains and population groups in community settings. From a health equity standpoint, an increasingly popular narrative is that RPM can: (1) enable remote care for marginalised groups including those in rural and remote areas^6^; (2) support older adults desiring to age in place^7–10^; and (3) support those experiencing poor access to care (e.g. unattached patients) by eliminating or reducing indirect health-related costs at individual and system levels^11,12^. However, significant individual, structural, and systemic barriers challenge the legitimacy of these claims; those who need it most, may not be able to experience the benefits of RPM^13,14^. Those most impacted by the digital health divide are usually structurally marginalised communities experiencing multiple forms of exclusion in access to health and social services^15^. Barriers to access include low digital literacy, low affordability of devices or internet data, and poor connectivity^16–18^. Furthermore, analysis reveals that digital health innovations (DHI) are more likely to perpetuate or introduce inequities^19^. For example, there is evidence of differential access to patient portals in racialized and marginalized communities compounded by age and race^20,21^, gender, geography, abilities, income, and digital (health) literacy^21^. Furthermore, many DHIs (e.g., electronic health records) require an internet connection, which inadvertently excludes by design, those who live in digital deserts^22,23^. In the United States up to 40% of low-income households lack an internet-subscription^22,24^. In Canada, when compared to the general public, fewer older adults (83% vs 94%) and rural residents (46% vs 87%) have access to the internet^25–27^. As such, internet access has been named a “super social determinant of health” because it impacts all other social determinants of health^18,22,28,29^. When these structural differences manifest within the context of RPM programs, it can increase digital and health equity divides through “intervention-generated inequalities” where there is a disproportionate benefit for those with existing social advantage at the expense of disadvantaged communities^30^. Thus, greater attention is required to avoid leaving structurally marginalised communities behind in the digital transformation era.

The aim of this rapid analytical review was to establish the extent to which RPM programs are positioned to advance equity through inclusive practices. Instead of an outcome-oriented approach- i.e. focusing on the extent to which RPM solutions improve health outcomes, we took a closer look at whether existing RPM interventions were positioned to advance equity. This approach extends the work of Woolley and colleagues who focused on equity of digital health technologies from a use and access perspective^31^. Our analysis includes articles from Australia, Canada, the United Kingdom, United States, and countries in Europe from the period 2017 to 2022 that report on RPM programs used to manage chronic diseases. We anticipated a mismatch between how RPM programs have been designed and implemented, and their projected or implied ability to advance equity^32,33^. We propound that unmasking assumptions regarding whom remote monitoring serves is a critical first step in ensuring no one is left behind as RPM is scaled up in different contexts.

## Methods

### Overview

As part of a large-scale provincial (in Ontario, Canada) evaluation of RPM programs for chronic diseases and COVID-19, our team was involved in a rapid literature review to identify and describe different models of technology-enabled RPM programs and to assess their impact on patient outcomes, patient experience, and provider experience. Articles identified during this parent review were streamlined (based on specific criteria outlined below) to conduct an equity analysis.

### Search databases

The parent review involved searching Medline and EMBASE databases to identify RPM programs, defined as those using a technology device to regularly measure a patients’ physiological or clinical data, and that involve remote transmission of those results either manually (patients completing electronic questionnaires) or automatically (e.g., Bluetooth or Wi-Fi linked device automated data transfer). The broad review focused on all relevant RPM papers globally between 2017 and 2022 that were used to support COVID-19 patients or those being managed for at least one of four commonly prevalent chronic diseases: health failure (HF), chronic obstructive pulmonary disease (COPD), diabetes (types 1, 2, and gestational), and hypertension (Supplementary Table 1). To inform the RPM search terms, we used a modified version of the Canadian Agency for Drugs and Technologies in Health (CADTH) telehealth search string^34^. KM (University of Toronto, information specialist) designed the search syntax, finalised, ran, and downloaded identified publications from both databases. The OVID Medline search was PRESS peer-reviewed by a second academic health sciences librarian before translation into each database (i.e., platform’s command language, including text words, controlled vocabulary, and subject headings). This yielded 27,248 relevant papers which were subsequently filtered to meet the objectives of the equity analysis.

### Inclusion criteria for equity analysis

We narrowed the focus of the equity analysis based on country and disease domain. The equity analysis was limited to English language articles from Australia, Canada, United States, and countries in Europe, including the United Kingdom for three reasons. First, most publications on RPM come from these countries. Second, these countries have had a relatively similar pace in advancements in digital health technology. Third, membership of many of these countries in larger groups (e.g., members of the European Union) is indicative of common aspirations and benchmarks in health care, including in relation to health equity. Multi-country papers that reported implementation sites outside of these countries of focus were also included in the equity analysis as they could not be disentangled.

### Exclusion criteria

**Fig. 1:**
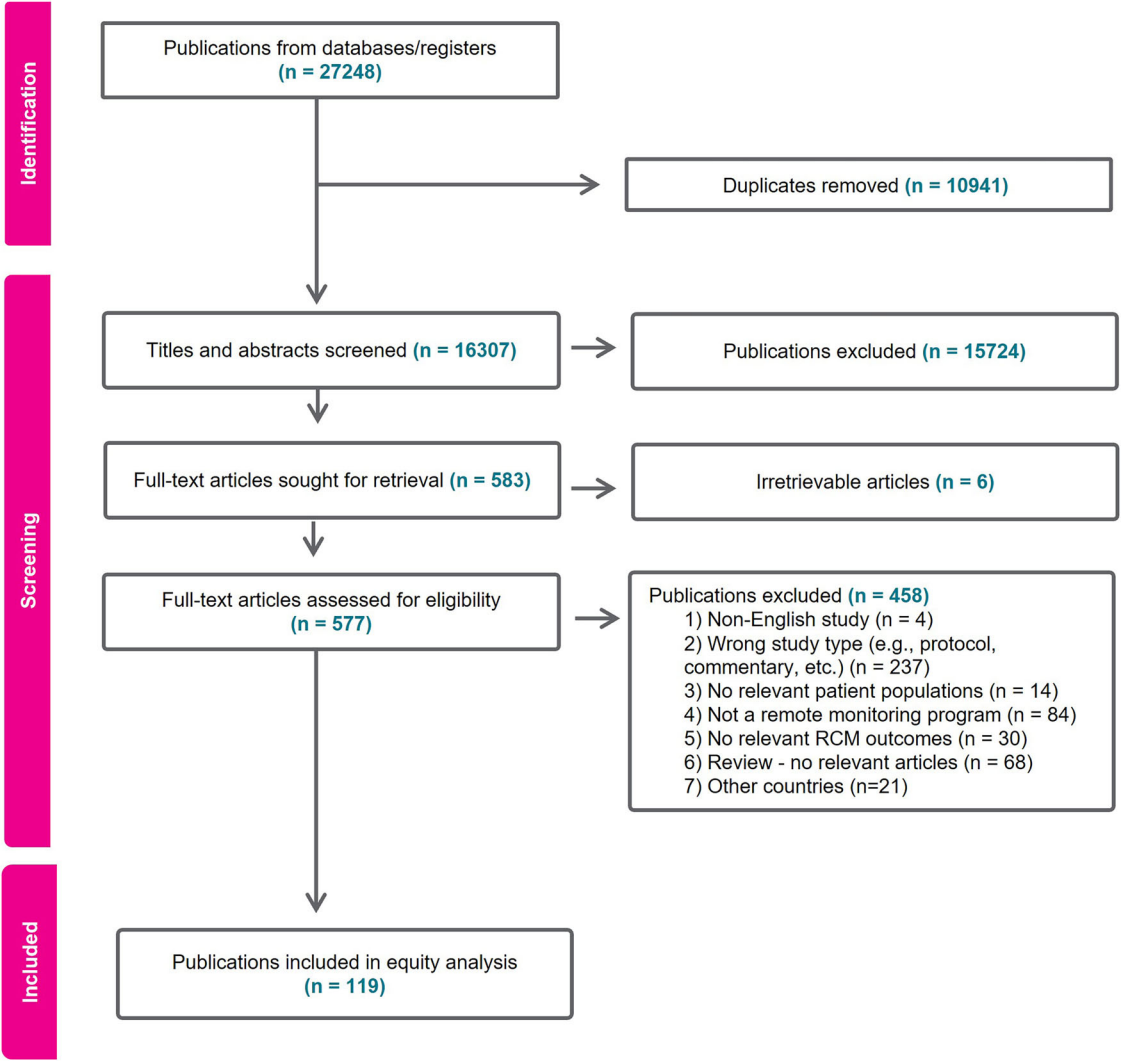
PRISMA diagram showing study identification for equity analysis. 119 publications were included from an initial 27,248 publications which were screened for eligibility based on title, abstract, and full article assessment.

We excluded commentaries, protocols, and reviews or meta-analysis because we sought to extract information on actual RPM programs. We excluded papers on COVID-19 since RPM as a model of digitally enabled care has advanced more in chronic disease management. We also excluded generic descriptive papers on RPM that did not describe the implementation of any specific intervention. This yielded a final inclusion of 119 papers. See PRISMA flow in Fig. 1.

### Guiding frameworks and data extraction

The parameters against which the equity analysis was conducted were informed by two established frameworks. The first guiding framework was the widely used PROGRESS PLUS framework developed by Cochrane^32^ for stratifying health opportunities and outcomes. PROGRESS refers to place of residence, race/ethnicity/culture/language, occupation, gender/sex, religion, education, socioeconomic status, and social capital, while PLUS refers to additional context-specific factors such as personal characteristics associated with discrimination (e.g., age, disability), features of relationships, and time-dependent factors (i.e., periods of temporary disadvantage such as immediately after hospital discharge). The second guiding framework was the digital health equity framework by Richardson et al.^33^, which outlines the digital determinants of health (DDoH) according to domains and levels of influence, mirroring the socioecological model^33^.

Using our experiences in health equity and RPM programs, we identified 11 equity-related parameters from both frameworks and used these as a guide to extract information from the 119 included articles (Supplementary Table 2). More specifically, we built a Macros-enabled data extraction template in Microsoft Excel^©^ (Microsoft Corporation, Redmond, USA). To ensure uniform categorization, each cell used drop down options and multiple options were selectable. We included an ‘Unknown’ option for each extraction category to capture missing data, as well as a free-text column to comment on additional information (e.g., specify race/ethnicity/culture). In addition to general information on each article (i.e., authors, year of publication and country of intervention), our extraction covered the following: level of care at which program was deployed (i.e., acute/hospital/specialized care versus home/primary/community care); chronic care disease domain (i.e., ‘COPD’, ‘Diabetes’, ‘CHF’ and ‘Hypertension’); and age (i.e., ‘0–15 years’, ‘16–64 years’, ‘65+ years’). Supplementary Table 2 includes the full extraction categories and descriptions.

### Parameters and datapoints for equity analysis

**Fig. 2:**
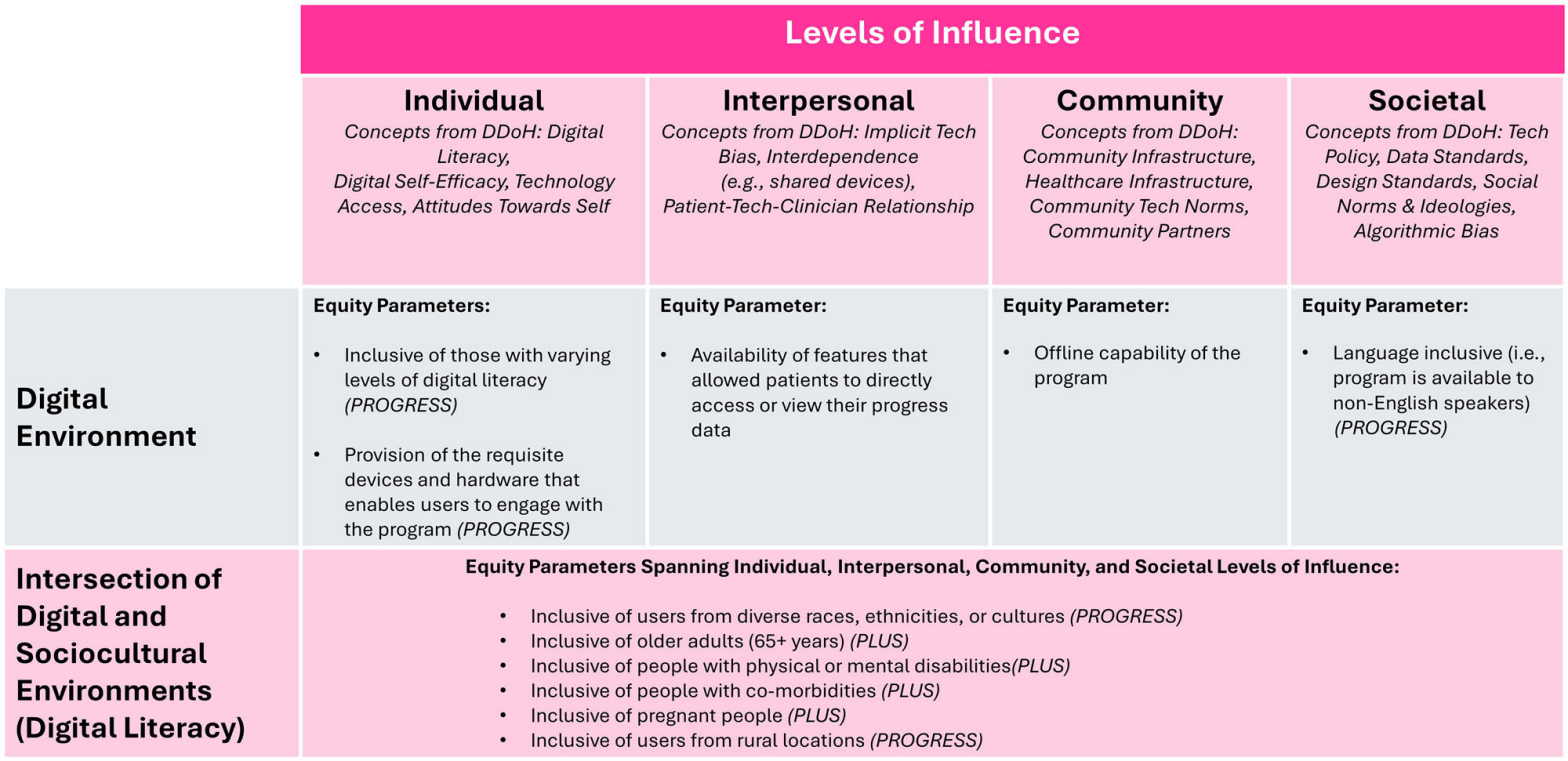
Equity parameters mapped against levels of influence in digital determinants of health (DDoH)^33^. We mapped five of the 11 equity parameters onto the digital environment, with the remaining 6 considered as cross-cutting parameters which span all levels of influence at the intersection of both the digital and sociocultural environments. Additionally, nine of the 11 parameters were directly mapped onto a *PROGRESS* or *PLUS* characteristic^32^.

We use the term parameter to describe a variable of interest (e.g., *country, age, geographic location*), and the term datapoint to represent instances within a parameter. For example, based on our inclusion criteria, all 119 papers reported on the extraction parameter *country*. However, there were 123 datapoints for this parameter, which reflects how several publications described multi-country RPM solutions. Using our extraction template, we sought to extract information to assess if the 119 papers reflected any of the 11 equity-related parameters, based on their reported inclusion criteria and program coverage. The 11 equity parameters were: (1) inclusive of users from diverse races, ethnicities, or cultures; (2) language inclusive (i.e., program is available to non-English speakers); (3) inclusive of people with physical or mental disabilities; (4) inclusive of people with co-morbidities; (5) inclusive of pregnant people; (6) inclusive of those with varying levels of digital literacy; (7) provision of the requisite devices and hardware that enables users to engage with the program versus bring your own device (i.e., technology provision); (8) offline capability of the program (i.e., does not require Internet connection); (9) availability of features that allowed patients to directly access or view their progress data; (10) inclusive of users from rural locations; and (11) inclusive of older adults (65+ years). Building off the work of Richardson and colleagues^33^ Fig. 2 outlines how each of these 11 equity parameters intersect with the DDoH^33^ and PROGRESS PLUS^32^ frameworks. At this time, gender and sex inclusivity were omitted to avoid further perpetuation of bias for three reasons. First, sex and gender tend to be used interchangeably despite representing discrete concepts (i.e., biological sex versus socially constructed roles, behaviours, expressions, and identities). Second, historical reporting standards are confounded by assumptions. For example, reporting that a sample was comprised of 60% males is meant to imply that 40% of the sample was female, but is not explicitly stated. Third, traditional binary categorization of sex and gender perpetuates harmful structural marginalization (e.g., questioning the place of intersex, non-binary persons).

### Equity parameter mapping

We conducted initial group discussions to ensure the categories and mapping process were similarly understood by the team. Quality assurance measures taken were as follows. Guided by a senior researcher- AI-O, three researchers (NU, KM, and VK) were responsible for mapping the equity parameters such that each of the 119 articles was independently color-coded by at least two researchers. Articles were mapped to equity parameters in batches of five to ten to ensure ongoing consistency of selection and extraction. In lieu of tracking interrater reliability, we maintained dual-researcher coding for all papers with group discussions occurring regularly to reconcile discrepancies when they arose. Validity was further enhanced through systematic tracking of rationale for exclusion (reflected in Fig. 1). Additionally, all articles received a quality assurance check by a third researcher who was not involved in the initial extraction^35^ and the team met regularly with AI-O throughout the process. This ensured a consistent analytical approach and regular reflection on findings. We did not conduct a quality assessment on any of the publications. Additionally, we did not explore program outcomes or their impact on equity or any other (clinical) outcomes, as doing so would have assumed the different programs were comparable or equity oriented de facto. Our aim was to document evidence in a descriptive and analytical format on the extent to which existing RPM programs were truly enabling pathways for equitable access.

## Results

### Overview

119 publications were identified for final inclusion in the equity analysis following deduplication and screening of articles against inclusion criteria (i.e., country, type of study, and disease domain). While 119 studies were included, the total number of datapoints (i.e., instances of occurrence) for each parameter (i.e., variable of interest) was affected by whether that parameter was reported at all, and the presence of multiple groups reported in a study. For example, some programs provided RPM services to groups under 65 years as well as those over 65 years of age, yielding multiple datapoints on the parameter of *age* in a single publication. For most parameters, the number of datapoints (N), matched the number of publications (*N* = 119). Exceptions included: country of intervention (*N* = 123); geographic location (*N* = 128); level of care (*N* = 131); disease domain (*N* = 137); age inclusivity (*N* = 187); and geographical region (*N* = 128).

#### Country

The 119 publications in the equity analysis included both single and multi-country programs (*N* = 123). Most RPM solutions were implemented in either the United States (43/123; 35%) or Europe (39/123; 32%), with proportionate representation across Australia (12/123; 10%), Canada (13/123;11%), and the United Kingdom (16/123; 13%).

#### Level of care

The 119 papers analyzed captured RPM available at single or multiple *levels of care* (*N* = 131). This parameter was well documented with 91% (119/131) data available. Across the 119 publications, the target group was most often for acute or specialized care (80/131; 61%) compared to primary, home, or community (39/131; 30%), or unknown settings (12/131; 9%).

#### Disease domain

The 119 papers analyzed included RPM solutions for at least one chronic disease domain, with some programs catering to multiple *disease domains* (*N* = 137). Nevertheless, disease domain was fully documented (100%) across all 119 RPM publications. Across the four chronic conditions of interest the proportion was accounted for as follows with the highest number targeting diabetes (45/137; 33%), followed by CHF (35/137; 26%), COPD (30/137; 22%), and hypertension (27/137; 20%).

### Reporting completeness of equity parameters

**Fig. 3:**
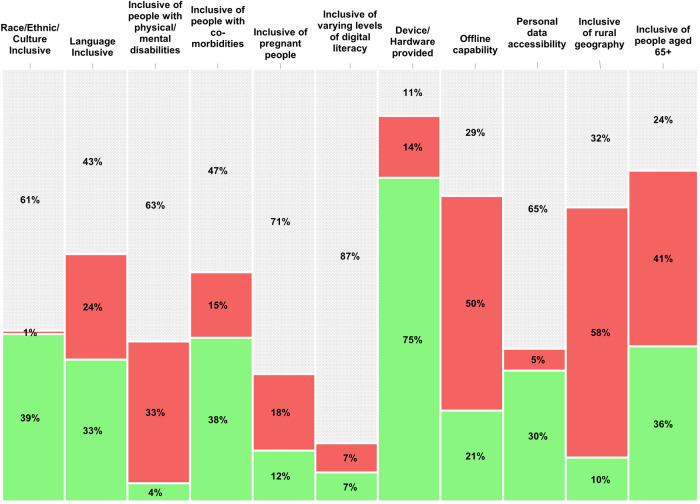
Equity analysis of 119 remote patient monitoring publications. 119 included remote patient monitoring articles represent Australia, Canada, the United Kingdom, the United States, and European countries. Columns represent each of the 11 equity parameters. Each column depicts proportion (%) of the 119 analysed papers that included (green; yes), did not include (red; no), or did not report (hashed grey; unknown) each equity parameter.

Generally, equity parameters were inconsistently and poorly reported (Table 1). We labelled such instances as “unknown” meaning we did not interpret these to mean exclusion, but rather non-reporting. Five (45%) of the 11 equity parameters had over 60% unknown data points across the 119 papers assessed. Specifically, data on inclusion related to race/ethnicity, disability, pregnancy, digital literacy, and data accessibility were more frequently unreported. Inclusion related to language and co-morbidity, accounted for over 40% of unknown variable points. The top two most reported equity parameters, internet access and technology provision, still had 29 and 11% incomplete reporting across all papers (see Fig. 3).

**Table 1:** Completeness of data reporting on 11 equity parameters (n) across 119 papers

Equity Parameter	Coverage of datapoints reporting:		
	Yes	No	Unknown
	*n* (%)	*n* (%)	*n* (%)
Race, ethnicity, or culturally inclusive?	46 (39%)	1 (1%)	72 (61%)
Language inclusive?	39 (33%)	29 (24%)	51 (43%)
Physical or mental disability inclusive?	5 (4%)	39 (33%)	75 (63%)
Co-morbidity inclusive?	45 (38%)	18 (15%)	56 (47%)
Pregnancy inclusive?	14 (12%)	21 (18%)	84 (70%)
Digital literacy inclusive?	8 (7%)	8 (7%)	103 (86%)
Technology provision inclusive?	89 (75%)	17 (14%)	13 (11%)
Internet access inclusive?	25 (21%)	59 (50%)	35 (29%)
Personal data accessibility?	36 (30%)	6 (5%)	77 (65%)
Geographical region inclusive (e.g., rural)	13 (10%)	74 (58%)	41 (32%)
Inclusive of older adults (e.g., 65+)	67 (36%)	76 (41%)	44 (23%)
**Average %**	**28%**	**24%**	**48%**

Parameter total count was 119 for 9 of 11 parameters. Due to the inclusion of multiple groups included within a single study, geographical region had a total count of 128 and inclusive of older adults had a total count of 187. For example, the first row will be read as 46 papers were inclusive of race, ethnicity or culture, 1 paper excluded certain groups, and in 61% of the papers, the inclusion/exclusion decisions for this parameter were unknown

Bold values present the averages.

### Equity analysis

We produced a visual equity heat map based on the analysis (Fig. 3). From an access perspective, most articles included provision of hardware (75%). However, only 30% provided users access to their own health data, and 58% of articles took place in urban areas (only 10% included rural locations). Regarding inclusion of structurally marginalized communities, less than half (36%) reported being available to older adults (people aged 65 and older) or being inclusive of people with diverse race/ethnicity/culture (39%), inclusive of people with comorbidities (38%), or language diversity (33%). For the publications where pregnancy status was reported, a larger proportion of papers excluded (18%) as opposed to included (12%) pregnant people. Most fundamentally, less than 10% of papers reported being inclusive of persons with varying levels of digital literacy (7%), or people with physical/mental disabilities (4%).

## Discussion

This paper represents empirical evidence that the widely held belief that RPM solutions generally advance equity and access to care for equity-deprived populations is closer to myth than reality. While it was surprising to see that almost 40% of programs in our analysis catered to older adults, the expected accessibility principles that guide AgeTech (i.e., digital technologies that support the health and wellbeing of older adults), such as digital literacy and offline capability were not areas where inclusion was prioritised. Although a main promise of RPM has been its ability to improve access to care for the most marginalised, we found that most papers did not explicitly report on the 11 equity parameters used in our analysis which represent common criteria for digital inclusion. A recently published article identified 30 high-priority digital determinants of health^18^. These include connectivity, device and software ability, digital literacy, health and disability status, inclusive and good practice design, socioeconomic inequalities, access and sharing policy^18^. As a complement, the concept of underinvested communities described by Holmes Fee et al., includes age, culturally and linguistically diverse backgrounds, urban environments, persons with chronic disease, abilities, race/ethnicity^36^. Underinvested communities that the priority health determinants and accompanying recommendations focus on are reflected in all 11 of our equity parameters^18,36^.

**Fig. 4:**
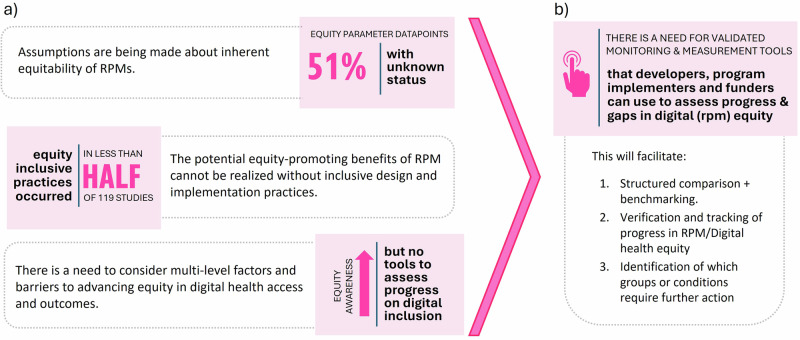
Summary of conclusions and recommendations. Panel (**a**) outlines three central takeaways; panel (**b**) outlines next steps and rationale.

Overall, what matters equity-wise in the field of DHI needs to move from anecdotal references to action at individual, interpersonal, community and societal levels of the DDoH. It is also a call to action at policy and programmatic levels, where accountability and reporting mechanisms can ensure systematic monitoring of if and to what extent digital health solutions like RPM are advancing health equity. We explore the central takeaways and recommend next steps (Fig. 4) in detail below.

In analysing RPM programs against equity parameters, we found incongruence between the current rhetoric on RPM and their theoretical or presumed potential to serve as inclusive access points to care. When equity parameters were reported, many programs in fact signaled *exclusion* (see Fig. 3). For example, less than half (39%) of publications were inclusive of race/ethnicity/and culture, only 9% of publications catered to rural residents, were inclusive of those with low digital literacy (8%), and far less (4%) were inclusive of those with physical or mental disabilities. Although RPM is seen as being beneficial for supporting the care needs of vulnerable patients, a rapid review on barriers of RPM use during COVID-19 highlights the converse^37^. RPM can inherently restrict access to its benefits through equity-related factors including ageism, cream skimming (whereby patients with complex needs are disadvantaged), geographic location, gender, racism, and socioeconomic status^16,38^. One salient example further exemplifying this paradox is in the domain of chronic diseases which have the most advanced RPM pathways, yet there is an under-representation in RPM implementation for minority groups^39^ even though they are disproportionately affected by chronic conditions (e.g., type 2 diabetes). Even when they are included, minority groups seem to experience diminished benefits and sub-optimal improvements^40^.

The bottom line is that while the potential for RPM is established, evidence of positive equity impact is not yet robust^41^. A report by Thompson et al., in the United States showed differential access to RPM favoring those living in urban settings and in census areas with lower social vulnerabilities^42^. This suggests that RPM programs are not being implemented with equity as a consideration for access and impact^22^. Without concerted and intentional effort, it is more likely that inequitable RPM solutions will remain normalized.

This equity analysis illustrated the high degree to which equitable design and access of RPM solutions are yet to be realised. The degree of missing datapoints, either from not reporting or having not been considered, hint at equity assumptions or inattention being made in the design, development, and implementation of RPM solutions. Additionally, for 10 of the 11 equity parameters, less than 40% of papers reported an inclusive practice, suggesting that not enough is currently being done and that inclusive practices are the exception, not the rule. This may also be due to under reporting as also experienced by Barcellona et al., in their framework to measure health equity in the Association of Southeast Asian Nations region^43^.

There is a need for technology vendors, payors, and implementation teams to critically appraise the extent to which RPM solutions are truly meeting the needs of those likely to benefit from them versus those who are most likely to be able to use them conveniently (i.e., designed for cherry picking). The dominant narrative which calls for expanding the use of remote monitoring solutions must be tempered by the realisation that prior to rapid scale, digital health equity needs to be prioritized and addressed. While the words quality, equity, and innovation have become interwoven bylines of advancements in RPM and digital technology in general, our results align with others^31,44^ in indicating that there is insufficient evidence to support those conclusions.

One additional factor is on how to balance equity considerations with context- or program-specific relevance. Including all 11 parameters in each study may be unrealistic and inappropriate for every context or program. For example, studies that are intentionally scoped to be narrow in nature by design do not need to address all 11 parameters before being considered equity-promoting (e.g., RPM for use in rural settings, by English speaking young adult populations). However, in these scenarios, we propound that standardized equity reporting on DHIs like RPM is necessary for understanding parameters that have been prioritised against others that are deemed to not apply. The developed checklist for reporting DHIs is a welcome step in the direction of systematic reporting, however it does not emphasize equity-related aspects^45^. Fundamentally, without standard guidelines and reporting tools, assumptions that are being made have the potential to inflict harm by excusing intervention-generated-inequalities.

Our findings aligns with the concern by Woolf and Vinson on how recruitment strategies are typically biased towards patients with higher cultural health capital^46^, i.e., those who possess the complement of relational, attitudinal, and technical skills required to maximise the benefits of clinical interactions and the care experience^47^. With reduced likelihood that those who experience higher rates of marginalization will have higher health capital, the risk of exclusion-by-design with RPM can easily introduce or reinforce inequities in health care access and by extension, outcomes of care. This ‘dark logic’^48^ state culminates in differential access to opportunities for involvement in a digitally driven health system, with fewer opportunities for equity-denied patients to both grow cultural health capital and engage meaningfully in decisions about their own care^46^.

Since our article search (up till March 2022), there has been increasing awareness of the need to situate and advance the goals of an equity promoting digital healthcare system^31,38,49,50^. While interest to address these gaps remains prominent in discourse, quantifying the nature and extent of exclusion can better inform the scale and spread of existing technologies, and inform an action plan for future initiatives. Additionally, structural and systemic factors are needed to robustly understand barriers to equitable adoption and uptake of RPM solutions^33,49^. In practice, this is being corroborated; in a 2023 paper on sex-and race-based disparities and opportunities in heart failure, Mastoris et al. identified several structural and systemic factors that explain the low referral and uptake of females and Black patients into RPM programs for heart failure, including socioeconomic, historical and technology-related factors^38^. They cited a need for multi-level interventions ingrained in policies that address the social determinants of health. This is one of the strengths of DDoH - we must evolve how RPM solutions are typically designed as standalone technological products, ignoring the broader social and policy systems with which they interact. To truly benefit a diverse range of individuals, digital health programs need to consider and be woven into the community and societal milieu^33,49^. This has implications for policymaking, design standards, and wireless infrastructure (society level), as well as community partnerships for planning sustainable access to technology beyond the duration of pilot studies (community level). Digital health equity will remain a myth without a closer look and commensurate response at the infrastructural and systemic contexts.

Currently, it is unclear how to operationalise digital health equity frameworks like DDoH across the RPM development pipeline. Guiding principles and frameworks exist, but there is much work to translate these into action. For example, at the design phase, human-centred or inclusive design practices have been identified as a crucial early step in promoting equitable digital health solutions^51^. But while equitable design considerations are necessary, they do not ensure RPM solutions are deployed as intended and in equitable ways, even if intended in theory. A mechanism for benchmarking progress is needed to drive efforts on standardized reporting and consideration of multi-level complexities and barriers that shape system-level impacts of RPM programs. This paper reinforces that standardized reporting can serve as an initial next step to facilitate benchmarking equitable RPM initiatives^43^. To track implementation-effectiveness progress, a validated, contextually relevant equity tool is needed. This tool would support program administrators and policymakers in systematically comparing and benchmarking RPM solutions, provide a mechanism for helping researchers understand where their RPM programs stand, and inform the development and evaluation of action plans to address equity. Our call to action for different actors in transforming how equity is considered in RPM programs is as follows:

- Policymakers: Invest in digital health technologies in a way that incentivizes equity. This means when considering how well an RPM program performed, there needs to be a weighting placed on equity (i.e., beyond simple counts of number of patients served). Inherently, prioritizing equity in performance evaluations will help scale programs that advance equity. It will also signal policy interest that stimulates how developers and implementers respond.
- Health service researchers and methodologists: Develop a tool to support policy makers in adopting equity as a consideration to guide investment analysis. This tool should not only include equity parameters but support the prioritization of which ones to focus on at a given point within a project’s lifecycle and as appropriate for the context. In so doing, it can facilitate monitoring and tracking of equity parameters within and across RPM programs over time. Such a tool may leverage the 11-equity parameters we have used here derived from *PROGRESS-PLUS*^32^ and DDoH^33^, or the health system framework to measure health equity^43^.
- Technology developers: Consider the economic value of costs saved and clinical benefits when a value-based care approach informs how RPM programs are developed for a given market or population. Given growing disparities in healthcare, developers are necessary partners in rethinking the design of RPM.
- Implementation teams: Detailed and transparent reporting that captures intervention intent, realised objectives and relevant equity indicators. Excluded or unaddressed equity parameters should be discussed within study limitations and where feasible, their projected impact on intervention-generated inequalities should be quantified.

### Limitations

The benefit of conducting a rapid analytical review is the ability to answer a targeted question in a timely manner. However, this approach presents some drawbacks. First, in the absence of a consistent way to compare effectiveness while accounting for other relevant factors, our review did not consider the impact or effectiveness of the RPM programs. Second, the use of a categorical approach (i.e., yes/no/unknown) did not capture if and how RPM users are engaged throughout the stages of design, development, implementation, and evaluation of RPM solutions. And third, aside from hardware provision, there was a minimum of 24% of datapoints with an unknown status. This makes it difficult to assess whether there was a true lack of inclusion in these programs, or whether there was a failure to report them. While we chose not to extract information based on sex and gender to avoid perpetuating conflated assumptions, it will be necessary to include these parameters in future analyses as reporting standards for sex and gender become more transparent and inclusive. Furthermore, we acknowledge that a requirement to include all 11 equity parameters is not feasible for all projects and some RPM programs may still be equitable with a narrow focus. Future studies should explore patient engagement across RPM stages, address reporting gaps in excluded equity parameters, and develop a framework to track equity within RPM programs over time. Additionally, future work should focus on establishing equity-oriented reporting guidelines.

## Conclusion

Our findings challenge the widely held belief that remote monitoring solutions equitably ensure the provision of health services for marginalised and underserved populations. Without challenging this notion, there is a risk of continued inaction and lack of intentional strategies to address the digital equity divide. In an age of rapid advancements in the use of technology to support access to and outcomes of healthcare, while equity has been central to the discourse, our analysis shows that remote patient monitoring initiatives are suffering from equity techno-solutionism- the belief that in and of themselves, remote technologies will address health equity. While we do not contend the potential of remote care, the expectations of what RPM can accomplish in minimizing or eliminating health inequities is still under scrutiny. Those who are most likely to benefit from the clinical and patient empowerment pathways that RPM promises to deliver are most frequently excluded from it and there is danger that assumptions on equity will unintentionally exacerbate the digital divide. Standardized reporting is needed with consideration for multi-level complexities and barriers. Furthermore, a contextually relevant and validated easy-to-use equity tool would help quantify efforts and facilitate benchmarking. This article aims to serve as a wake-up call to technology developers, health system planners, and innovation implementers that equity is not a by-product of technology but rather requires concerted intentional action to ensure inclusivity.

## Supplementary information

Supplementary Information

## Data Availability

The paper includes all relevant information as depicted in the table and figures. Additional data can be provided upon request. Please direct your inquiries to Dr. Ibukun Abejirinde (ibukun.abejirinde@thp.ca).
