# Supplementary material for: An equity analysis of remote patient monitoring programs unveils assumptions on digital health equity
Source: NPJ Digit Med. 2025 May 29;8:320. doi: 10.1038/s41746-025-01731-x (PMC12122910; doi:10.1038/s41746-025-01731-x)
Supplement: Supplementary file 1 — Supplementary Information [file 41746_2025_1731_MOESM1_ESM.pdf]

Supplementary Information

Supplementary Table 1. Medline Search Syntax

#	Searches	Results	Type
1	Remote Consultation/ or exp Telemedicine/ or Remote Sensing Technology/	42883	Advanced
2	((computer or distance or internet or phone or online or remote or tele* or video or virtual or web) adj2 (administ* or advice or assess* or care or chat* or confer* or consult* or counsel* or deliver* or health* or interv* or manag* or medic* or monitor or nurs* or pharm* or therap* or visit*)).ti,ab,kf.	99800	Advanced
3	(remot* adj4 monitor*).tw,kf.	5864	Advanced
4	(teleadminist* or teleassess* or telecare or telechat* or teleconf* or teleconsult* or teledeliv* or telehealth* or teleinterv* or telemanag* or telemedic* or telemonit* or telenurs* or telepharm* or televisit* or teletherap* or videochat* or videotelephon* or videoconsultation or videophone* or wireless tech* or telecardiology or telehypertension or smart device or smart phone or electronic consultation or telediagnosis or telepathology).ti,ab,kf.	34882	Advanced
5	(eConsult* or e-consult* or eHealth* or e-Health* or einterv* or e-interv* or etherap* or e-therap* or mHealth* or m-Health* or mobile health* or Mobile application*).ti,ab,kf.	24301	Advanced
6	Telemed*.jw.	6562	Advanced
7	exp Mobile Applications/	9689	Advanced
8	(App or apps or facetime* or skype* or zoom or webbased tool or web-based tool* or voice-over or voiceover or VoIP).ti,ab,kf.	42275	Advanced
9	or/1-8 [Remote monitoring]	194481	Advanced
10	exp lung diseases, obstructive/	226599	Advanced
11	(aecb or chronic airflow disease* or chronic airflow disorder* or chronic airflow limitation* or chronic airway disease* or chronic airway disorder* or chronic airway limitation* or chronic obstructive airflow disease* or chronic obstructive airway disease* or chronic obstructive airway disorder* or coad or cobd or copd or emphysema*).tw,kf.	80946	Advanced
12	((chronic* or persistent) adj3 bronchiti*).tw,kf.	11587	Advanced
13	(obstruct* adj3 (pulmonary or lung* or airway* or airflow* or bronch* or respirat*)).tw,kf.	97872	Advanced
14	or/10-13 [COPD]	294419	Advanced
15	exp Diabetes Mellitus, Type 2/ or Diabetes, Gestational/ or exp Diabetes Complications/ or Diabetes Mellitus/ or Diabetes Insipidus/ or Diabetes Mellitus, Type 1/	442769	Advanced
16	Diabetic Ketoacidosis/	7056	Advanced

17	(diabet* or NIDDM or IDDM or prediabet* or MODY or T1DM or T2DM or T1D or T2D or non insulin* depend* or noninsulin* depend* or noninsulindepend* or non insulin?depend*).tw,kf.	718962	Advanced
18	or/15-17 [diabetes]	775233	Advanced
19	exp Heart Failure/	136166	Advanced
20	(decompensation cordis or myocardial decompensation or chronic heart failure or chronic cardiac failure).tw,kf.	18001	Advanced
21	((Heart or cardiac or myocardial) adj2 (failure or chronic or decompensation or congestive)).tw,kf.	218071	Advanced
22	((left ventricular or left ventricle) adj2 (failure or insufficien* or dysfunction*)).tw,kf.	23536	Advanced
23	((dilated or congestive) adj2 cardiomyopath*).tw,kf.	20122	Advanced
24	((ventricular or ventricle*) adj2 (failure or insufficien* or dysfunction*)).tw,kf.	33857	Advanced
25	lvsd.tw,kf.	611	Advanced
26	or/19-25 [heart failure]	281251	Advanced
27	exp Hypertension/ or Blood Pressure/ elevated blood pressure/	509349	Advanced
28	(hypertens* or prehypertens*).tw,kf.	480942	Advanced
29	((blood or arterial or diastolic or systolic) adj3 pressure).tw,kf.	401371	Advanced
30	((elevat$ or increas$ or lower or high or rais$ or rising) adj2 (bp or dbp or hbp or sbp)).tw,kf.	15907	Advanced
31	or/27-30 [hyper tension]	878249	Advanced
32	COVID-19/ or exp COVID-19 Testing/ or COVID-19 Vaccines/ or SARS-CoV-2/	149778	Advanced
33	(coronavirus/ or betacoronavirus/ or coronavirus infections/) and (disease outbreaks/ or epidemics/ or pandemics/)	40110	Advanced
34	(nCoV* or 2019nCoV or 19nCoV or COVID19* or COVID or SARS-COV-2 or SARSCOV-2 or SARS-COV2 or SARSCOV2 or SARS coronavirus 2 or Severe Acute Respiratory Syndrome Coronavirus 2 or Severe Acute Respiratory Syndrome Corona Virus 2).ti,ab,kf,nm,ot,ox,rx,px.	229256	Advanced
35	((new or novel or "2019" or Wuhan or Hubei or China or Chinese) adj3 (coronavirus* or corona virus* or betacoronavirus* or CoV or HCoV)).ti,ab,kf,ot.	56136	Advanced
36	(longCOVID* or postCOVID* or postcoronavirus* or postSARS*).ti,ab,kf,ot.	31	Advanced
37	((coronavirus* or corona virus* or betacoronavirus*) adj3 (pandemic* or epidemic* or outbreak* or crisis)).ti,ab,kf,ot.	11732	Advanced
38	((Wuhan or Hubei) adj5 pneumonia).ti,ab,kf,ot.	387	Advanced
39	or/32-38	240063	Advanced
40	limit 39 to yr=2019 -Current [COVID]	238595	Advanced
41	14 or 18 or 26 or 31 or 40	2220236	Advanced
42	9 and 41	30977	Advanced
43	Animals/ not (Animals/ and Humans/)	4943573	Advanced
44	42 not 43	30641	Advanced
45	limit 44 to (english and last 5 years)	22533	Advanced

Supplementary Table 2. Categories used to extract information from included articles.

	Category/Options	Additional Notes/Description
A	Title	
B	Authors (Year)	
C	Country	
	- Canada	
	- United States	
	- Australia	
	- United Kingdom	
	- Europe	Excluding UK
D	Geographical location	
	- urban	Areas with a population of 50,000 +
	- rural	Areas with a population of less 50,000
	- both	
	- unknown	
E	Level of care	
	- primary/home/community care	Community care setting
	- acute/specialized care	Hospital setting
	- both	
	- unknown	
F	Disease domain	
	- COPD	
	- Diabetes	
	- CHF	
	- Hypertension	
G	Race/culture/ethnic inclusivity	
	- yes	Includes non-white groups.
	- no	All study participants were white.
	- unknown	
H	If yes, please specify	If answered yes to question G, please specify the race/culture/ethnicity listed.

I	Language inclusivity	
	- yes	Includes languages other than English.
	- no	Only English speakers included.
	- unknown	
J	Gender and sex inclusivity	
	- male	Study sample includes males.
	- female	Study sample includes females.
	- other (trans/bi/two-spirit/gender fluid…)	Study sample includes people that identify as other than male and female.
	- unknown	
K	Age inclusivity	
	- 0-15	Study sample include participants 0-15 years old.
	- 16-64	Study sample include participants 16-64 years old.
	- 65 +	Study sample include participants 65+ years old.
	- unknown	
L	Inclusive of ppl with physical/mental disabilities	
	- yes	If people with varying physical/mental capabilities were included in the study sample.
	- no	If people with varying physical/mental capabilities were included in the study sample.
	- unknown	
M	Inclusive of ppl with co-morbidities	
	- yes	If people with other co-morbidities were included in the study sample.
	- no	If people with other co-morbidities were excluded from the study.
	- unknown	
N	Inclusive of pregnant people	
	- yes	If pregnant people were included in the study sample.
	- no	If pregnant people were excluded from the study.
	- unknown	
O	Inclusive of varying levels of digital literacy	
	- yes	All participants included regardless of previous familiarity with techonology.

	- no	Eligible participants needed a baseline of skills with using technology.
	- unknown	
P	Device/Hardware provided	
	- yes	If devices needed were fully provided by the study facilitators.
	- no	If study required participants to have specific devices (not provided).
	- unknown	
Q	Offline capability	
	- yes	If there was offline capability and wifi/data was not required all the time.
	- no	If wifi/data were required all the time.
	- unknown	
R	Personal data accessibility	
	- yes	If participants could see and review their personal data on RPM over time.
	- no	If participants do not have access to their personal data.
	- unknown	
S	Notes	
T	Additional references	Please include the complete reference(s) for any supplementary articles that are related to the extracted article (ex: details other parts of the study, presents other data…)
